# OTUB1 accelerates hepatocellular carcinoma by stabilizing RACK1 via its non-canonical ubiquitination

**DOI:** 10.1007/s13402-023-00913-7

**Published:** 2024-02-05

**Authors:** Liqun Peng, Tiangen Wu, Yingyi Liu, Dongli Zhao, Wenzhi He, Yufeng Yuan

**Affiliations:** 1 Department of Hepatobiliary and Pancreatic Surgery, Zhongnan Hospital of Wuhan University, Wuhan, China; 2 Clinical Medicine Research Center for Minimally Invasive Procedure of Hepatobiliary and Pancreatic Diseases of Hubei Province, Wuhan, China; 3 College of Life Sciences, Hubei Key Laboratory of Cell Homeostasis, Wuhan University, Wuhan, China

**Keywords:** Hepatocellular carcinoma, OTUB1, RACK1, Ubiquitination, PI3K/AKT signaling pathway

## Abstract

**Background:**

Dysregulated ubiquitination modification occupies a pivotal role in hepatocellular carcinoma (HCC) tumorigenesis and progression. The ubiquitin aldehyde binding 1 (OTUB1) was aberrantly upregulated and exhibited the pro-tumorigenic function in HCC. However, the underlying mechanisms and responsible targets of OTUB1 remain unclear.

**Methods:**

First, bioinformatics analysis, western blot and immunohistochemistry staining were applied to analyze OTUB1 expression in HCC specimens. Then, immunoprecipitation assay-tandem mass spectrometry (MS) combined with the gene set enrichment analysis (GSEA) was used to explore the downstream target of OTUB1. Co-immunoprecipitation and ubiquitination assays were used to identify the mechanisms involved. Finally, we explored the regulatory effect of MAZ on OTUB1 through ChIP-qPCR and dual-luciferase reporter assay.

**Results:**

OTUB1 was broadly elevated in HCC tissues and promoted the proliferation and metastasis of HCC in vitro and in vivo. The receptor for activated C kinase 1 (RACK1) performed as a functional partner of OTUB1 and its hyperactivation was associated with aggressive development and other malignant features in HCC by activating oncogenes transcription. Mechanistically, OTUB1 directly bound to RACK1 at its C-terminal domain and decreased the K48-linked ubiquitination of RACK1 through its non-canonical suppression of ubiquitination activity, which stabilized RACK1 protein levels in HCC cells. Therefore, OTUB1 significantly increased multiple oncogenes expression and activated PI3K/AKT and FAK/ERK signaling in a RACK1-dependent manner in HCC. Moreover, the transcription factor MAZ upregulated OTUB1 expression through identifying a putative response element of OTUB1 promoter area.

**Conclusions:**

Our findings might provide a new therapeutic strategy for HCC by modifying the MAZ-OTUB1-RACK1 axis.

**Supplementary Information:**

The online version contains supplementary material available at 10.1007/s13402-023-00913-7.

## Introduction

Primary HCC was the sixth most common cancer and ranked third in terms of mortality rate among all tumors in 2020 [1]. Surgical resection is the recommended and most effective treatment for early-stage HCC while is no longer applicable as most HCC patients are diagnosed with advanced stages. The benefits of interventional therapy and local chemoembolization are limited for patients with advanced HCC [2, 3]. Therefore, a better understanding of molecular mechanisms behind HCC carcinogenesis and progression is imperative for the development of therapeutic targets with high efficacy. Hyperactivation of oncogenes expression is one of the hallmarks of HCC and is well-recognized as a promised therapy target [4–7]. Previous reports have indicated that RACK1 levels are abnormally upregulated in HCC and stimulate multiple oncogenes translation in a ribosome-bound form [8, 9]. Unfortunately, regulators of RACK1 expression have barely been identified in HCC.

Aberrant ubiquitination is the basic characteristic of HCC and proved to occupy an important role in HCC [10, 11]. Previously, we have reported that the ubiquitin-specific protease (USP) family members USP5 and USP53 act as oncogenes or tumor suppressor genes in HCC via the stabilization of c-Myc and cytochrome c (CYCS), respectively [12, 13]. OTUB1 is the deubiquitinase OTU superfamily member and participates in multiple pathophysiological processes, including immunological response, ferroptosis, cancer development, and so on [14, 15]. In multiple myeloma, OTUB1 promoted tumor survival by removing the K48-linked polyubiquitination chain from c-Maf to facilitate the transcription of oncogenes [16]. In breast cancer, OTUB1 stabilized MYC and increased HK2 expression which promoted aerobic glycolysis eventually [17]. In renal cell carcinoma, OTUB1 activated ECT-2-Rho signaling through the deubiquitination of FOXM1, which accelerated tumor growth [18]. Nevertheless, the molecular mechanism and potential substrates of OTUB1 in HCC remain elusive.

In the present study, we concentrate on the function of OTUs in HCC and identify OTUB1 as a crucial contributor to the malignant proliferation and metastasis of HCC. We investigated OTUB1 functioned as a tumor promoter in HCC through the deubiquitination of RACK1, which subsequently activated the downstream PI3K/AKT and FAK/ERK signaling pathway. The transcription factor MAZ accounts for OTUB1 abnormal expression in HCC. To sum up, our investigation suggested that MAZ-OTUB1-RACK1 axis is a novel promised therapeutic strategy for HCC.

## Materials and methods

### Bioinformatics

Transcriptomic data and clinical characteristics for HCC patients were gathered using the Cancer Genome Atlas (TCGA) database (https://portal.gdc.cancer.gov/). The following four microarray datasets were obtained from the Gene Expression Omnibus (GEO) database: GSE14520, GSE76427, GSE57957, and GSE105130. Additionally, R software (version 4.2.2) was used to examine the mRNA expressions of OTUB1.

### Cell culture and clinical specimens

Normal hepatocytes (THLE-2) were obtained from Meisen Cell Technology Co., Ltd (Zhejiang, China). HepG2, Hep3B, Huh7, Li7, and PLC/PRF/5 cell lines were obtained from the Cell Bank of the Chinese Academy of Sciences (Shanghai, China). MHCC9H and HCCLM3 cell lines were obtained from iCell Bioscience Inc (Shanghai, China). Cytogenetically testing and STR analysis were performed on each cell line to determine its identity within 2 years. The culture environment of the normal and tumor cells was as previously described [12]. Fresh HCC tumor tissues and paired normal tissues were obtained from Zhongnan Hospital of Wuhan University and with appropriate patient consent. Tissue samples were paraffin-embedded or frozen in liquid nitrogen and then stored at −80 °C.

### RNA interference, RNA isolation, and real-time PCR

The siRNA oligonucleotides corresponding to the target sequence of OTUB1 were designed and synthesized by GenePharma (Shanghai, China). The lentiviruses carrying shRNA against OTUB1 and the negative control were obtained from Genechem (Shanghai, China). All siRNA and shRNA sequences used in the present study were listed in Supplementary Table S1. TRIzol reagent (Invitrogen, CA, USA) was used to lysate and extract the total RNA of cell lines. The reverse transcription and amplification reaction were conducted using HiScript II Q Select RT SuperMix (#R233, Vazyme) and SYBR qPCR Master Mix (#Q711, Vazyme). Then, the mRNA expression of target genes was detected in the CFX96TM Real-Time System (Bio-Rad, California, USA). The specific primers used for qRT-PCR were provided in Supplementary Table S2.

### Plasmid construction and cell transfections

The Flag-OTUB1 full-length, 1-85 aa and 47-271 aa plasmids, Flag-OTUB1-D88A plasmid, Flag-OTUB1-C91S plasmid, His-RACK1 full-length and its deletion constructs were designed and manufactured by Sino Biological (Beijing, China). The GST-RACK1 plasmid with pGEX-3X vector and His-OTUB1 plasmid with pET28a vector were designed and manufactured by GENECREATE (Wuhan, China). The HA-K48, -K63, and -Ub plasmids were obtained from Genechem (Shanghai, China). DNA sequencing verified all constructions. All transfections were performed using JetPRIME^®^ (Polyplus-transfection S.A, Illkirch, France) according to the manufacturer’s instructions.

### Antibodies and reagents

Antibodies utilized in this work were shown in Supplementary Table S3. MK2206 (HY-108232), PD98059 (HY-12028), MG132 (HY-13259), and cycloheximide (HY-12320) were purchased from MCE (Shanghai, China). DMSO was purchased from Sigma (St. Louis, MO, USA, D2560).

### Western blotting analysis

RIPA lysate mixed with protease inhibitors (Beyotime Biotechnology, Shanghai, China) was used to extract cellular or tissue proteins. Following SDS-PAGE electrophoresis, the protein bands were transferred to the PVDF membrane, which was then sealed with 5% skimmed milk powder for 1 h. Then primary antibody was added and incubated overnight at 4 °C; the membrane was washed three times with TBST, HRP-labeled secondary antibody was added and incubated for 2 h at room temperature, then the membrane was washed three times with TBST, and the target protein expression level was detected by an ECL developing system (Tianneng).

### Co-IP and LC-MS/MS

Briefly, plasmid-transfected cells are lysed with IP buffer (20 mM Tris-HCl, pH 7.4; 150 mM NaCl, 1 mM EDTA pH 8, 1% NP-40, 1 × Protease, and Phosphatase Inhibitor Cocktail), and the protein was extracted by centrifuging the supernatant at 4 °C, 12,000*g*, for 10 min. The total protein concentration in the supernatant was assayed using a BCA kit (Beyotime, P0010S). The protein suspension was then incubated with 20 μl Protein A/G Magnetic beads (MCE, Shanghai) for 30 min to remove non-specifically bound proteins. Next, 20 μl new Protein A/G Magnetic beads and IP level antibodies were incubated overnight at 4 °C according to the standard of 2 μg antibody/1 mg total protein. The beads were then collected with a magnetic frame and washed three times with high-salt buffer (20 mM Tris-HCl, pH 7.4; 300 mM NaCl; 1 mM EDTA, pH 8; 1% NP-40) for 5 min each time. The magnetic beads were then washed three times with low-salt buffer (20 mM Tris-HCl, pH 7.4; 150 mM NaCl, 1 mM EDTA, pH 8, 1% NP-40) for 5 min each time. And the appropriate amount of 1 × SDS-loading buffer was added to boil the magnetic beads at 96 °C for 10 min. Finally, the boiled liquid was centrifuged at 4 °C, 12,000*g*, for 2 min and the supernatant was taken for subsequent experiments. SDS-PAGE gel electrophoresis was performed, and after Komas Brilliant Blue staining and decolorization, the gel was cut off and analyzed by mass spectrometry by Biotree Biotech (Shanghai, China).

### GST-pulldown assays

Bacterial-expressed His-OTUB1 fusion protein and GST-RACK1 fusion protein were combined in an equal amount (0.5 mg) and incubated on ice for 3 h. The mixture was then placed onto Glutathione Sepharose 4B resin columns. Proteins were eluted with wash buffer supplemented with 15 mM reduced glutathione after being washed five times with wash buffer. The eluates were transferred to PVDF membranes, separated using 12% SDS-PAGE, and then probed with the appropriate antibodies. As negative controls, GST and His from Genecreate (Wuhan, China) were employed. For each pull-down test, three replications were performed.

### Luciferase, and ChIP assay

For luciferase assay, cells were transfected with pGL3-OTUB1 WT or MUT and pGL3-Renilla with or without MAZ plasmids for 48 h. After lysing the cells, the Dual-Luciferase Reporter kit (Promega, Germany) was used to measure firefly luciferase activity, which was normalized by the Renilla luciferase luminescence data. Chip assay was performed with a ChIP assay kit from Millipore (Billerica, USA) as previously described [19]. Cells were preserved with formaldehyde, and DNA was sonicated to fragments between 100 and 500 bp. Then, the supernatants were flipped in an overnight incubation at 4 °C with antibodies against MAZ or regular serum IgG. A DNA purification kit was used to extract the eluted product, and the purified DNA was then employed as the PCR template. The primers were listed in Supplementary Table S1.

### Immunohistochemical (IHC) staining and immunofluorescence

For IHC, samples were fixed with 4% paraformaldehyde (PFA) overnight before paraffin embedding and sectioning into 4-μm thick sections. After dewaxing and rehydration, the slides were heated with citrate buffer or EDTA solution to extract tissue antigens. Primary antibodies were incubated with samples for an overnight period at 4 °C before the addition of the proper biotinylated secondary antibodies. Sections were then analyzed using a diaminobenzidine (DAB) chromogenic kit and counterstained with hematoxylin. For immunofluorescence, cells were fixed in 4% PFA for 15 min and permeabilized with 0.5% Triton X-100 for 30 min. After being blocked with goat serum for 1 h, cells were incubated with primary antibody overnight at 4 °C. Cells were then stained with secondary antibody (Servicebio, China) for 1 h at room temperature and with DAPI (Servicebio, China) for 5 min. Images were photographed with a confocal fluorescence microscopy system Microscopy System (Leica, Germany).

### Proliferation, invasion, and colony formation assay

The HCC cell invasion, proliferation, and colony formation experiments were carried out as previously described [12].

### Flow cytometry

2 × 10^5^–1 × 10^6^ HCC cells were harvested and washed once with PBS. After centrifuging the cell suspension, the supernatant was discarded. Then cell cycle analysis was performed using a cell cycle staining kit (CCS012, MultiSciences, China) following the manufacturer’s instructions. The proportion of cells in different cell cycles was measured with a CytoFLEX flow cytometer (Beckman, China). The data was saved and analyzed using FlowJo 10.0 software.

### In vivo ubiquitination assay

HCC and HEK293T cells were transfected with indicated plasmids along with or without HA-Ub for 48 h and then were treated with 10 μM proteasome inhibitor MG132 for an additional 8 h before being collected for co-immunoprecipitation. The ubiquitinated RACK1 was detected using an anti-HA antibody by immunoblotting.

### In vivo tumorigenesis and metastasis assay

6-to-8-week-old male BALB/c nude mice were acquired from Vital River Laboratories (Beijing, China) and kept in specialized pathogen-free environments at the Laboratory Animal Facility of Zhongnan Hospital. To establish HCC xenograft models, subcutaneous injections of HCCLM3 cell suspensions (5 × 10^6^ cells) with or without OTUB1 stable knockdown were administered to each group of mice (n = 5 in each group). The tumor volume was estimated by the formula length × width 2 × 1/2. The mice were sacrificed when the largest tumors reached a size of roughly 1000 mm^3^, and the tumors were then excised to determine the weight of the growth before being photographed. For the HCC lung metastasis model, 1 × 10^6^ HCCLM3 cells with or without OTUB1 stable knockdown were diluted in 100 μl of sterile PBS and injected through the tail vein. For the orthotopic liver tumor model, the right lobe of the liver was exposed through incision, and 3 × 10^6^ luciferase-labeled above-mentioned cells were injected. Images were captured using an IVIS 100 Imaging System (Xenogen, Hopkinton, MA, USA) 10 min after the injection of 150 mg/kg of D-luciferin (Goldbio, USA) intraperitoneally into the mice.

### Statistical analyses

Student’s *t*-test and one-way ANOVA were used to compare two and more groups respectively. Multiple comparison with Bonferroni correction was performed when appropriate. Prism 8.0 (GraphPad, USA) was used for all statistical analyses and a *p* value < 0.05 was taken as statistical significance.

## Results

### OTUB1 was upregulated in HCC tissues and correlated with poor prognosis

To identify HCC-related OTUs, we firstly checked the RNA-seq and clinical data from the TCGA database combined with the Kaplan–Meier Plotter (https://kmplot.com/analysis/). The detailed hazard ratio (HR), 95% confidence interval (CI), and log-rank *p* value were presented in Supplementary Table S4. Among them, we noticed that 5 OTUs (OTUD3, OTUB2, OTUB1, OTUD7B, and OTUD1) were linked to an increased risk of HCC (HR > 1 and *p* < 0.05) (Fig. S1A). Expression heatmap indicated OTUB1 changed mostly between HCC tumor and normal tissues compared with the other 4 OTUs (Fig. S1B–C). Consequently, OTUB1 was selected for further investigation.

**Fig. 1:**
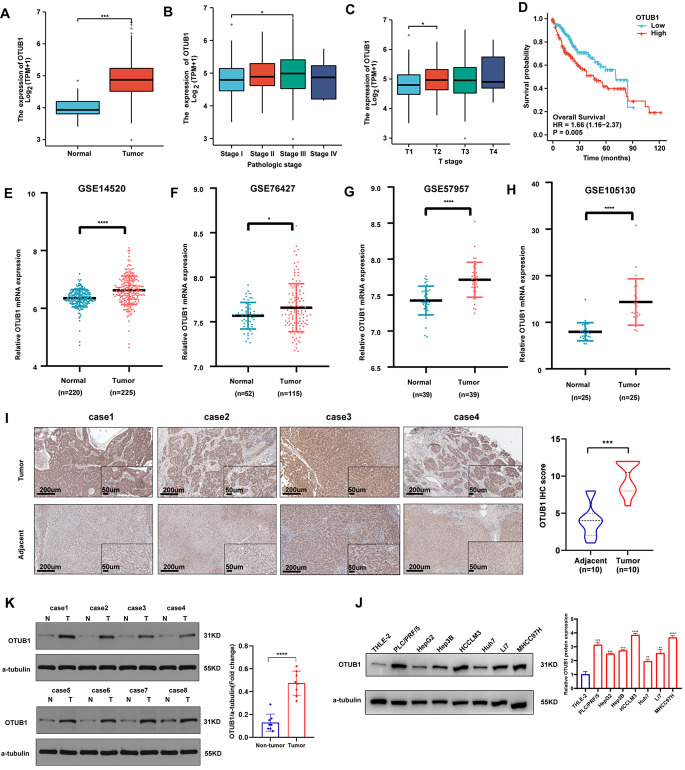
OTUB1 was elevated in HCC and associated with poor prognosis. **a** The mRNA expression of OTUB1 in HCC tumor samples (n = 371) and adjacent non-tumor samples (n = 50) in the TCGA database. **b, c** The mRNA expression of OTUB1 in HCC tumors of different pathologic stages (Stage I, n = 168; Stage II, n = 84; Stage III, n = 82; Stage IV, n = 6) and T stages (T1, n = 54; T2, n = 173; T3, n = 118; T4, n = 12). **d** OS rates for the HCC groups with different OTUB1 levels were displayed on Kaplan–Meier curves. **e–h** OTUB1 transcript abundance in HCC patients from the GEO database. **i** Representative IHC images of OTUB1 protein expression in 10 pairs of HCC and adjacent normal tissues. Scale bars (left), 200 μm. scale bars (right), 50 μm. **j** Western Blot showing OTUB1 protein levels in 8 pairs of HCC and peri-tumor tissues. **k** Western Blot showing OTUB1 expression in 7 HCC cell lines and normal hepatocytes (THLE-2). *p < 0.05; **p ≤ 0.01; ***p ≤ 0.001; ****p < 0.0001

OTUB1 was significantly elevated in HCC tumors compared to the normal liver tissues (Fig. 1a). OTUB1 expression level was positively correlated to pathologic and tumor stages (Fig. 1b, c) and negatively correlated to overall survival (Fig. 1d). Consistently, four GEO HCC datasets supported elevated tumoral OTUB1 mRNA levels as well (Fig. 1e–h). Furthermore, IHC staining and WB revealed that the OTUB1 protein expression was obviously higher in the malignant tissues than in the non-cancerous tissues in our HCC patient samples (Fig. 1i, j). In comparison to the normal liver cell line THLE-2, HCC cell lines also exhibited higher levels of the OTUB1 protein (Fig. 1k). In summary, these findings indicated that OTUB1 was upregulated in HCC and that its overexpression was associated with malignant progression and poor prognosis.

### OTUB1 significantly promoted the proliferation and migration of HCC cells in vitro

**Fig. 2:**
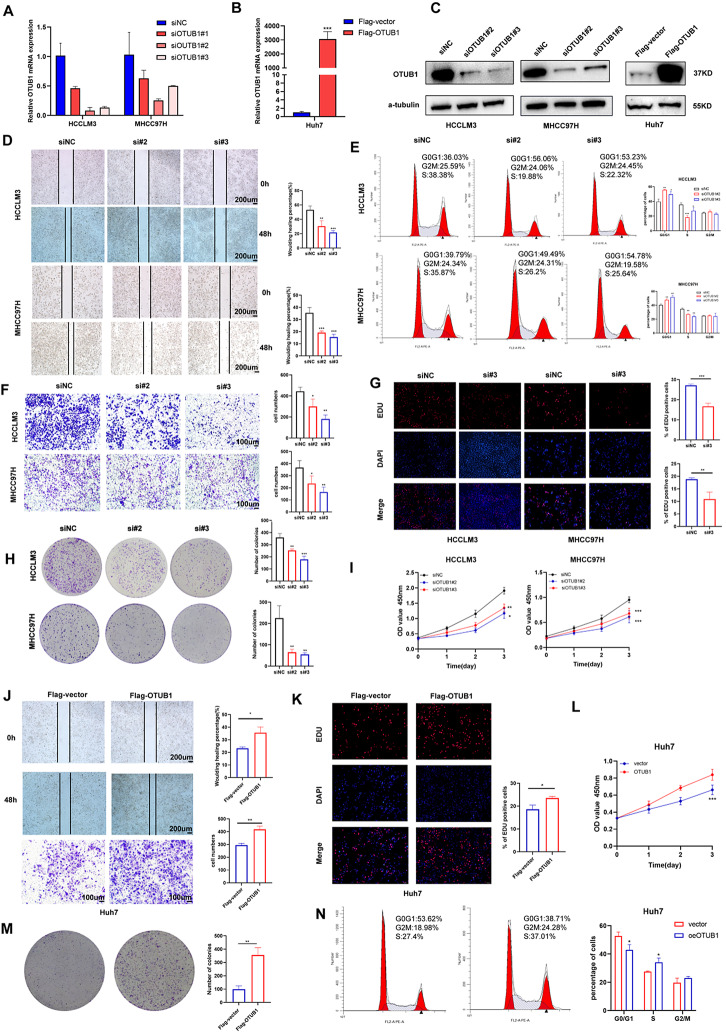
OTUB1 accelerated the proliferation and metastasis of HCC cells in vitro. **a** RT-PCR analyses for OTUB1 knockdown efficiency in HCCLM3 and MHCC97H cells. **b** RT-PCR analyses for OTUB1 overexpression efficiency in Huh7 cells. **c** Western blot analyses for the transfection efficiency of the above-mentioned cells. **d** Wound healing assays were utilized to evaluate the migration ability of the control and OTUB1-disrupted HCCLM3 and MHCC97H cells. **e** Flow cytometry assays were utilized to evaluate the cell cycle distribution of the control and OTUB1-disrupted HCCLM3 and MHCC97H cells. **f** Transwell assays were conducted to assess the invasion ability of the control and OTUB1-disrupted HCCLM3 and MHCC97H cells. **g** EDU staining assays were used to detect the proliferation percentage of the control and OTUB1-disrupted HCCLM3 and MHCC97H cells. **h, i** Colony formation and CCK8 assays of the control and OTUB1-disrupted HCCLM3 and MHCC97H cells. **j** Wound healing assays and transwell assays demonstrating the migration and invasion capacity of Huh-7 cells transfected with vector or Flag-tagged OTUB1 plasmids. **k–n** EDU staining assays, CCK8, colony formation, and flow cytometry assays demonstrating the proliferation capacity of Huh-7 cells transfected with vector or Flag-tagged OTUB1 plasmids. All experiments were carried out three times. *p < 0.05; **p ≤ 0.01; ***p ≤ 0.001; ****p < 0.0001

To elucidate whether OTUB1 participates in HCC progression, we modulated OTUB1 expression levels in vitro. RNA interference was performed in MHCC97H and HCCLM3 cells and OTUB1 was overexpressed in Huh7 cells. qRT-PCR and WB were employed to validate the transfection efficiency (Fig. 2a–c). OTUB1 deficiency inhibited the migration and invasion capacity of MHCC97H and HCCLM3 cells (Fig. 2d, f), and decreased the proliferation, colony-formation abilities, and distribution of the S phase (Fig. 2e, g–i). Accordingly, OTUB1 overexpression dramatically raised the proliferative, colony-forming, and migrating abilities of Huh7 cells (Fig. 2j–n). Collectively, these results supported that OTUB1 overexpression is the indicator of poor HCC prognosis.

### Effect of OTUB1 on HCC growth and metastasis in vivo

**Fig. 3:**
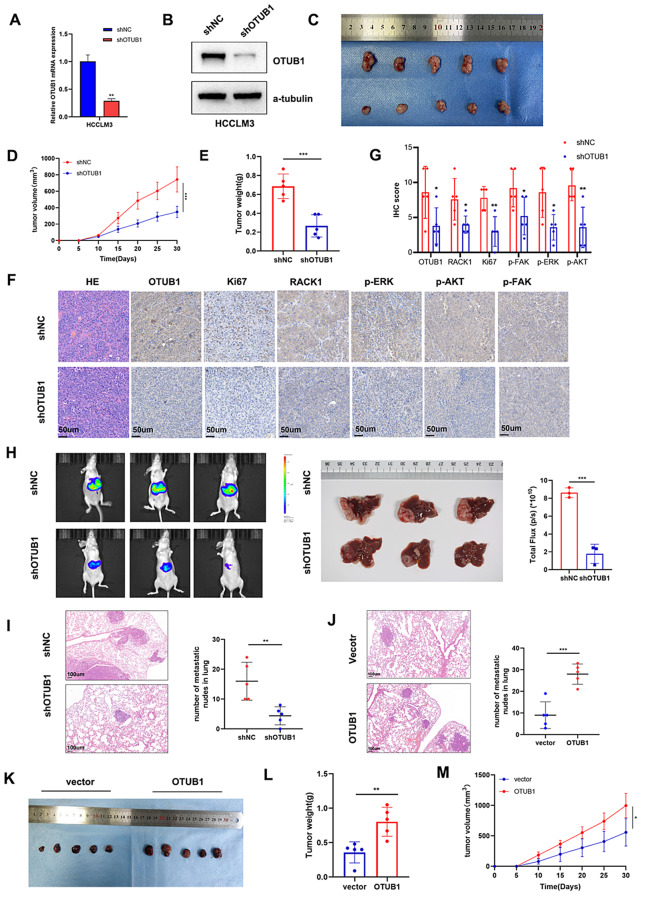
OTUB1 silencing inhibited HCC tumor growth and metastasis in multiple mice models. **a, b** Stable OTUB1 knockdown efficacy was verified by qPCR and WB assays. **c–e** Tumor image, tumor volume, and tumor weight of HCCLM3 tumor xenografts stably expressing either shNC or shOTUB1 (n = 5 mice/group). **f** The protein levels of OTUB1, Ki-67, RACK1, p-ERK, p-AKT, and p-FAK in the xenograft tumors were assessed by IHC. Scale bars, 50 μm. **g** Quantitative IHC scoring of the xenograft tumors (n = 5). **h** Representative bioluminescent images of orthotopic liver tumor model. The indicated cells were orthotopically inoculated in the livers of nude mice (n = 3 mice/group). **i** HE staining of lung metastatic tumors obtained from nude mice injected with shNC or shOTUB1 HCCLM3 cells (n = 5 mice/group). Scale bars, 100 μm. **j** HE staining of lung metastatic tumors obtained from nude mice injected with lv-vector or lv-OTUB1 Huh7 cells (n = 5 mice/group). Scale bars, 100 μm. **k–m** Tumor image, tumor weight, and tumor volume of Huh7 tumor xenografts stably expressing either lv-vector or lv-OTUB1 (n = 5 mice/group). *p < 0.05; **p ≤ 0.01; ***p ≤ 0.001

To further validate the roles of OTUB1, the xenograft HCC tumor model was constructed. 10 male BALB/c nude mice were equally divided for subcutaneously injection with OTUB1-disrupted (shOTUB1) and control (shcontrol) HCCLM3 cells (n = 5 mice/group). Successful OTUB1 knockdown was confirmed by qPCR and WB analysis (Fig. 3a, b). When sacrificing, the tumor volumes and weights indicated that OTUB1 knockdown greatly repressed the subcutaneous tumor formation (Fig. 3c–e). And IHC staining of OTUB1, KI67, RACK1, p-ERK, p-AKT, and p-FAK supported that efficient OTUB1 knockdown ameliorated the hyperactivation of the oncogenic pathway (Fig. 3f, g), and corroborated the reduced subcutaneous tumor growth when OTUB1 silenced. Furthermore, in orthotopic HCC mouse models, the OTUB1 knockdown group displayed notably slower tumor growth (Fig. 3h). Figure S2C showed the representative H&E staining images of the liver tumor volume in each group. Next, we examined the potential involvement of OTUB1 in tumor metastasis in vivo. The lung metastasis rate was dramatically reduced in the shOTUB1 group in comparison to the shcontrol group, as demonstrated in Fig. 3i. Conversely, increased lung metastasis rates were observed when OTUB1 was overexpressed (Fig. 3j). The huh7 xenograft tumor models further showed that the stable overexpressed-OTUB1 group represented a greater tumor volume and weight than the control group (Fig. 3k–m). And IHC staining showed compared to the control group, a relatively elevated OTUB1, KI67, RACK1, p-ERK, p-AKT, and p-FAK expression were detected in the OTUB1-overexpressed group (Fig. S2A–B). Collectively, these findings Illustrated OTUB1 stimulated HCC growth and metastasis in vivo as indicated in vitro.

### OTUB1 directly interacted with and increased the expression of oncoprotein RACK1

**Fig. 4:**
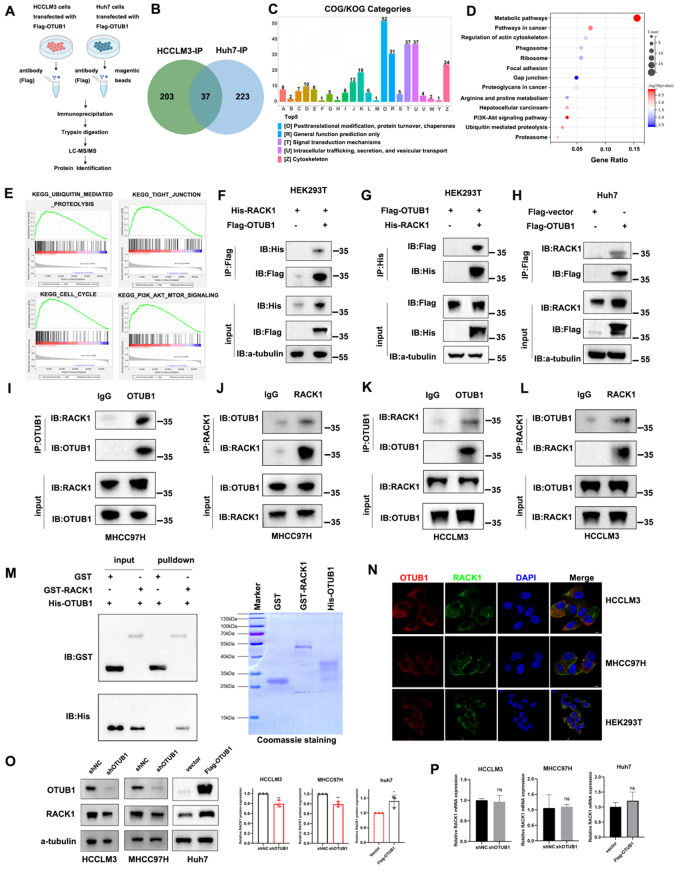
OTUB1 directly interacted with and increased the expression of oncoprotein RACK1 in HCC. **a** The LC-MS/MS strategy was shown in the flowchart. **b** Venn diagram of LC-MS/MS results showing 37 co-interacted proteins. COG class annotation (**c**) and KEGG pathway analysis (**d**) of all identified proteins by LC-MS/MS. **e** GSEA was performed between the high and low OTUB1 expression groups to further explore OTUB1-related signaling pathways in HCC. **f, g** The exogenous connection between OTUB1 and RACK1 was determined utilizing co-IP and western blotting assay in HEK293T cells. **h** Coimmunoprecipitation of OTUB1 and RACK1 in huh7 cells transfected with Flag-empty/Flag-OTUB1 plasmids. **i–l** The endogenous interaction between OTUB1 and RACK1 was detected by co-IP and western blotting assay in MHCC97H and HCCLM3 cells. **m** OTUB1 and RACK1 directly interacted with each other as demonstrated by GST pull-down assays. **n** OTUB1 (red) and RACK1 (green) colocalization via immunofluorescence in HCCLM3, MHCC97H, and HEK293T cells. DAPI (blue) was used as a counterstain for nuclei. **o** Immunoblotting and **p** qPCR assays of RACK1 expression in shOTUB1-treated HCCLM3, MHCC97H cells, and OTUB1-overexpressed Huh7 cells. *p < 0.05; **p ≤ 0.01; ***p ≤ 0.001

To investigate the mechanism behind the HCC-enhancing effects of OTUB1, we adopted mass spectrum detection for OTUB1-interacted proteins in HCCLM3 and Huh7 cells (Fig. 4a). 240 proteins in HCCLM3 cells and 260 proteins in Huh7 cells were identified respectively, which are potential OTUB1 interactors (Supplementary Tables S5 and S6) (Fig. 4b). All detected proteins in the IP-MS data were functionally annotated using Clusters of Orthologous Groups (COG) analysis, which revealed a significant proportion of proteins were involved in signal transduction and posttranslational modification pathways (Fig. 4c). Top15 KEGG enriched pathways of potential interacting proteins were associated with pathways in cancer, regulation of actin cytoskeleton, focal adhesion and gap junction, etc. (Fig. 4d). To narrow the range of candidate interacting proteins, we analyzed the common regulators of OUTB1 surrogates in TCGA-LIHC database through the gene set enrichment analysis (GSEA). The OTUB1-high expression group displayed significant elevation of several cancer-promoting pathways, including CELL_CYCLE, TIGHT_JUNCTION, and PI3K_AKT_MTOR_SIGNALING (Fig. 4e). Among these putative targets of OTUB1, we concentrated on receptor for activated C kinase 1 (RACK1) in our current study, considering its important roles in hepatocellular carcinogenesis and exacerbating CRC via activating PI3K-AKT pathway [9, 20]. To validate the potential key role of RACK1 in OTUB1-mediated tumor suppression, we performed the exogenous Co-IP assay first. Results showed that RACK1 is combined with OTUB1 in 293T cells (Fig. 4f, g). Moreover, the endogenous Co-IP experiment confirmed that RACK1 has interaction with OTUB1 in HCC cells (Fig. 4h–l). Additionally, the GST-pull-down assay demonstrated their direct interaction (Fig. 4m). Immunofluorescence labeling and confocal imaging also indicated that OTUB1 and RACK1 are mainly co-localized in the cytoplasm of both HCC cells and HEK293T cells (Fig. 4n). We further explored whether OTUB1 has any impact on the expression and stability of RACK1 in HCC cells after confirming the direct connection between them. Our data indicated that shRNA-mediated OTUB1 knockdown reduced the quantity of RACK1 protein and that OTUB1 overexpression increased RACK1 protein in HCC cells (Fig. 4o). As expected, the RACK1 mRNA level was not substantially changed despite silencing or overexpressing OTUB1 in HCC cells (Fig. 4p).

### OTUB1 reduced K48-linked ubiquitination of RACK1 and stabilized RACK1 via its non-canonical ubiquitination

**Fig. 5:**
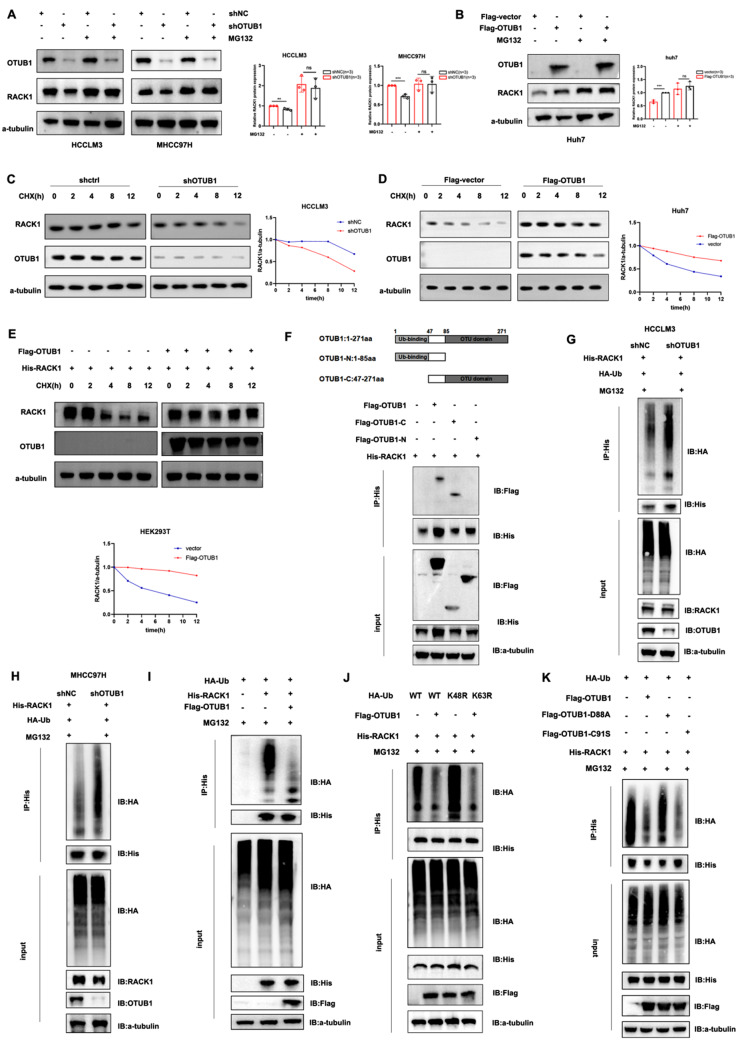
OTUB1 stabilizes RACK1 by decreasing the K48-linked ubiquitination of RACK1. **a** The control and OTUB1-depleted HCC cells (HCCLM3 and MHCC97H) were treated with or without 10 µM MG132 for 8 h and then harvested for immunoblotting. The graphs displayed the results of quantitative analyses of RACK1 protein levels. **b** Huh7 cells were transfected for 48 h with either the Flag-OTUB1 plasmid or vehicle before being treated with 10 μM MG132 for an additional 8 h. RACK1 protein level was then measured by immunoblotting. Quantitative analyses were shown in the graphs. **c** The control and OTUB1-disrupted HCCLM3 cells were exposed to 100 μg/ml CHX for the indicated time and then collected for immunoblotting. **d** Huh7 cells were transfected with vector or Flag-OTUB1 plasmids for 48 h and exposed to 100 μg/ml CHX for the indicated time before being collected for immunoblotting. **e** HEK293T cells were transfected with the indicated plasmids for 48 h and exposed to 100 μg/ml CHX for the indicated time before being collected for immunoblotting. **f** HEK293T cells were transfected with the His-RACK1 plasmids along with the truncated variants of OTUB1 for 48 h. Co-IP assay was then conducted to identify the OTUB1 and RACK1 interaction domains. **g, h** MG132 (10 µM) was applied to the control and OTUB1-depleted HCC cells (HCCLM3 and MHCC97H) for 8 h after co-transfecting with His-RACK1 and HA-Ub plasmids. Later, the RACK1 ubiquitination level was detected by IP and western blot. **i** HEK293T cells were co-transfected with Flag-OTUB1, HA-Ub, and His-RACK1 for 48 h and applied with MG132 (10 µM) for 8 h before harvest. Later, the RACK1 ubiquitination level was detected by IP and western blot. **j** HEK293T cells were co-transfected with different ubiquitin mutants, Flag-OTUB1 and His-RACK1 for 48 h. Later, cells were exposed to 10 μM MG132 for 8 h, and then western blot and IP were performed. **k** HEK293T cells were co-transfected with His-RACK1, HA-Ub, together with Flag-OTUB1, Flag-OTUB1-D88A, or Flag-OTUB1-C91S for 48 h. Later, cells were exposed to 10 μM MG132 for 8 h, and then western blot and IP were performed

We tried to verify whether increased RACK1 expression is proteasome-dependent considering OTUB1 is a deubiquitinase. As shown in Fig. S3A, the proteasome inhibitor MG132 could increase the protein level of RACK1, indicating that RACK1 can be degraded by the ubiquitin-proteasome system (UPS). In addition, MG132 treatment significantly reversed the down-regulation of RACK1 in OTUB1-depleted MHCC97H and HCCLM3 cells (Fig. 5a). Consistently, OTUB1 overexpression raised the RACK1 protein level in Huh7 cells, whereas MG132 treatment minimized this effect (Fig. 5b). HCC cells were treated with cycloheximide (CHX), a protein synthesis inhibitor, to determine whether OTUB1 altered RACK1 stability. The half-life of RACK1 was dramatically decreased in OTUB1-depleted HCC cells (Fig. 5c), while significantly lengthened in HCC cells that overexpressed OTUB1 (Fig. 5d, e). All these data demonstrated that OTUB1 deubiquitinates RACK1 and improves its stability by inhibiting ubiquitin-proteasome pathway-mediated degradation.

Then, a series of domain-deleted mutants of OTUB1 and RACK1 were constructed for further research. Co-IP assay indicated the C-terminal of OTUB1 was responsible for mediating the interaction with RACK1 (Fig. 5f). Additionally, we discovered that the RACK1 mutant with its WD2–WD4 domain deleted was unable to engage with OTUB1, indicating this interaction was carried out through its WD2–WD4 domain (Fig. S3B).

Subsequently, we investigated if OTUB1 might deubiquitylate RACK1. As indicated in Fig. 5g, h, OTUB1 deficiency significantly catalyzed RACK1 ubiquitination in HCCLM3 and MHCC97H cells. Meanwhile, in vitro ubiquitination assay showed OTUB1 overexpression notably reduced RACK1 ubiquitination in HEK293T cells (Fig. 5i). Following that, we investigated which lysine (K)linked polyubiquitin chains of RACK1 might be eliminated by OTUB1. When we simultaneously mutated K48 in the ubiquitin molecule to arginine (HA-Ub K48R), we found OTUB1 mediated ubiquitination of RACK1 was eliminated (Fig. 5j). Next, to determine the specific ubiquitination sites of RACK1, we generated a series of lysine (K) to arginine (R) mutants of RACK1 (K106R and K127R) based on previous findings that O-GlcNAcylation at Ser122 was involved in the modulation of RACK1 protein stability and prevents its ubiquitination [8]. Our results showed that OTUB1-mediated ubiquitination of RACK1-K127R was significantly abolished, suggesting that the K127 residue of RACK1 was a key ubiquitination site (Fig. S3C). Previous studies have shown that OTUB1 regulates the deubiquitination process through two distinct mechanisms: the typical enzymatic activity for polyubiquitin hydrolysis and the atypical activity for the generation of E2 inhibitory complexes [15, 21]. We designed two functional mutants of OTUB1 with different activities, C91S (disrupts deubiquitination enzyme activity) and D88A (disrupts the interaction with E2-binding enzymes). CO-IP assays showed both two mutants could also interact with RACK1 similar to that of wildtype OTUB1 (Fig. S3D–E). Co-transfected RACK1 protein level was not stabilized by overexpressing OTUB1-D88A, whereas it was stabilized by overexpressing OTUB1-C91S in HEK293T cells (Fig. S3F). The in vitro ubiquitination assay also proved overexpression of OTUB1 (C91S) effectively promoted the stability of RACK1, while OTUB1 (D88A) was ineffective (Fig. 5k). These findings suggested that the binding between OTUB1 and RACK1 is not dependent on its deubiquitinating enzyme activity, but may be related to its interaction with E2-binding enzymes. However, the exact mechanism of OTUB1-induced RACK1 stabilization needs to be further elucidated.

### Inhibition of AKT and ERK signaling blocked OTUB1-induced aberrant activation

**Fig. 6:**
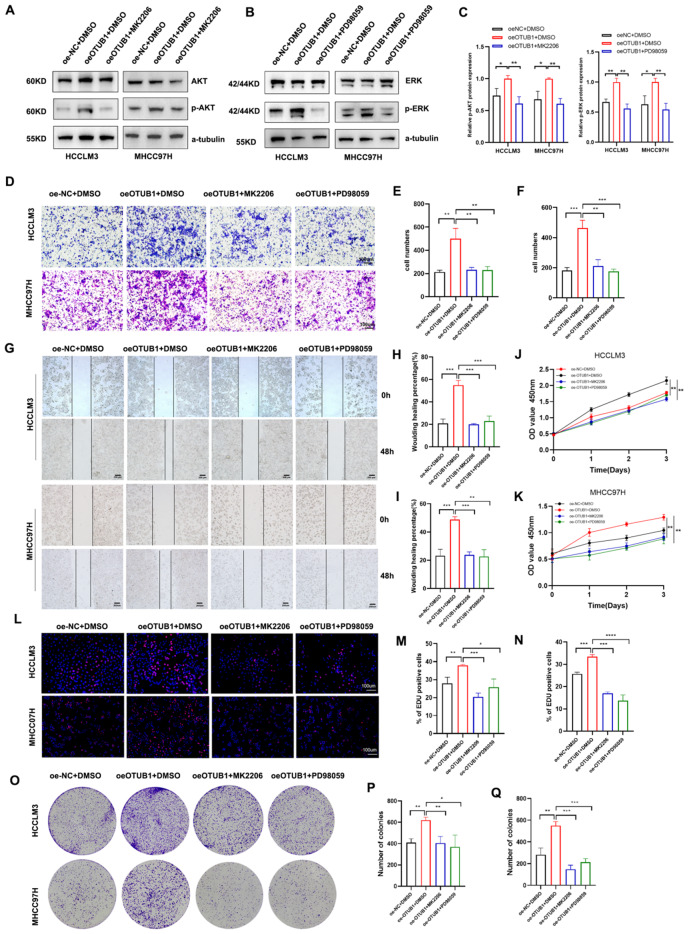
Inhibition of AKT and ERK signaling blocked OTUB1-induced aberrant activation. **a, b** HCCLM3 and MHCC97H cells were transfected with Flag-OTUB1 or mock control, followed by further treatment with MK2206 (2 μM) and PD98059 (20 μM) for 48 h. Western blot was conducted to detect the protein levels of p-AKT and p-ERK. **c** ImageJ v1.8 software was used to quantify protein abundance. **d–f** Transwell assays revealed the invasion ability of these transfected HCC cells. **g–i** Wound healing assays revealed the invasion ability of these transfected HCC cells. **j, k** CCK-8 assay revealed the proliferation ability of these transfected HCC cells at 0, 24, 48, and 72 h. **l–q** EDU assays and colony formation assays revealed the proliferation ability of these transfected HCC cells. Data represent means ± SEM. *p < 0.05, **p < 0.01, ***p < 0.001

RACK1 was reported to exert its multiple functions in malignancies via activating its downstream oncogenic signaling. To clarify the downstream effectors of OTUB1 in HCC cells, we investigated a number of potentially implicated signaling. Western-blot assays indicated that overexpression of OTUB1 notably enhanced the activation of AKT and ERK signaling pathways (Fig. 6a–c), which is consistent with previous reports [22, 23]. Besides, when we blocked PI3K/AKT and/or MEK/ERK signaling by its specific inhibitor MK2206 and PD98059, the promotive effect of OTUB1-induced malignant behavior of HCC cells was also significantly reversed (Fig. 6d–q), demonstrating that OTUB1 performed the tumor-enhanced effects by effectively activating the AKT and ERK signaling pathway.

### OTUB1 contributed to HCC progression by activating PI3K/AKT and FAK/ERK signaling in a RACK1-dependent manner

**Fig. 7:**
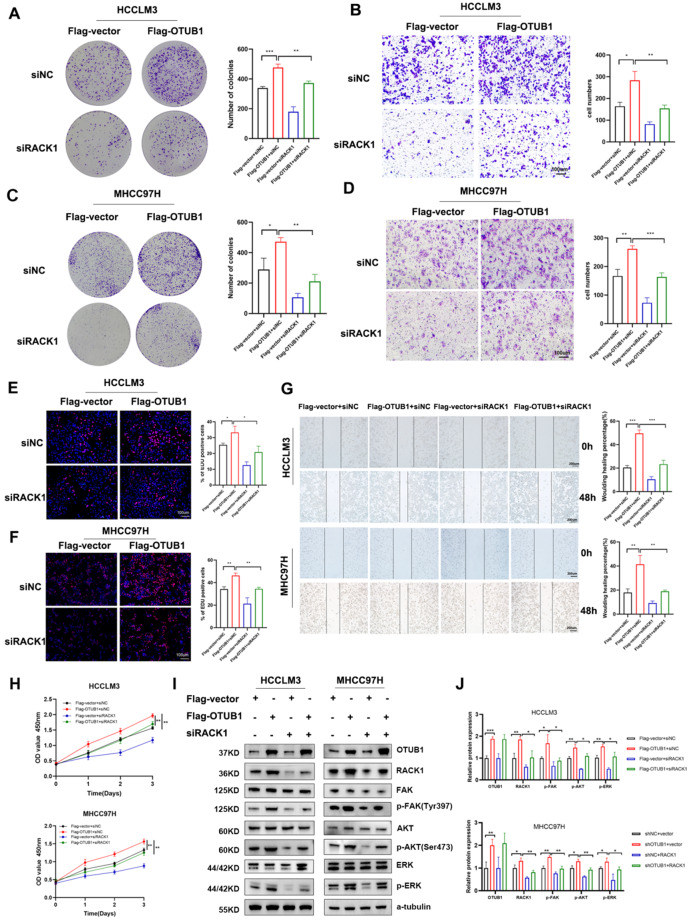
OTUB1 regulated PI3K/AKT and FAK/ERK signaling by promoting RACK1 in HCC cells. **a** Colony formation of HCCLM3 cells treated as indicated. **b** Transwell assays of HCCLM3 cells treated as indicated. **c** Colony formation of MHCC97H cells treated as indicated. **d** Transwell assays of MHCC97H cells treated as indicated. **e, f** EDU assays were performed to detect the proliferation of these transfected HCC cells. **g** Wound healing assays were conducted to detect the invasion of these transfected HCC cells. **h** CCK-8 assays demonstrated the proliferation viability of HCC cells treated as indicated. **i** HCC cells were co-transfected with Flag-OTUB1 plasmid and siRACK1 or control for 48 h before detecting the protein levels of FAK, p-FAK, AKT, p-AKT, ERK, and p-ERK in the indicated groups. **j** The protein abundance was quantified by ImageJ v1.8 software. Data represent means ± SEM. *p < 0.05, **p < 0.01, ***p < 0.001

To determine whether OTUB1 accelerated the development of HCC by modulating RACK1 expression, we loaded siRACK1 into the OTUB1-overexpressed HCC cells and detected the characteristics then. Results showed the aggressive malignant behaviors generated by OTUB1 overexpression were drastically reversed after lowering the RACK1 levels in HCC cells. Colony-formation assay, EDU assay, and CCK8 assays indicated the suppression of RACK1 partly decreased the HCC cell proliferation caused by OTUB1 overexpression (Fig. 7a, c, e, f, h). Moreover, the knockdown of RACK1 reversed the overexpression of OTUB1-induced migration and invasion of HCC cells (Fig. 7b, d, g). Immunoblotting revealed that the upregulation of p-AKT (Ser473), p-ERK (Thr202/Tyr204), and p-FAK (Tyr397) protein levels caused by OTUB1 overexpression were suppressed after RACK1 downregulation (Fig. 7i, j).

### MAZ promotes OTUB1 transcription

**Fig. 8:**
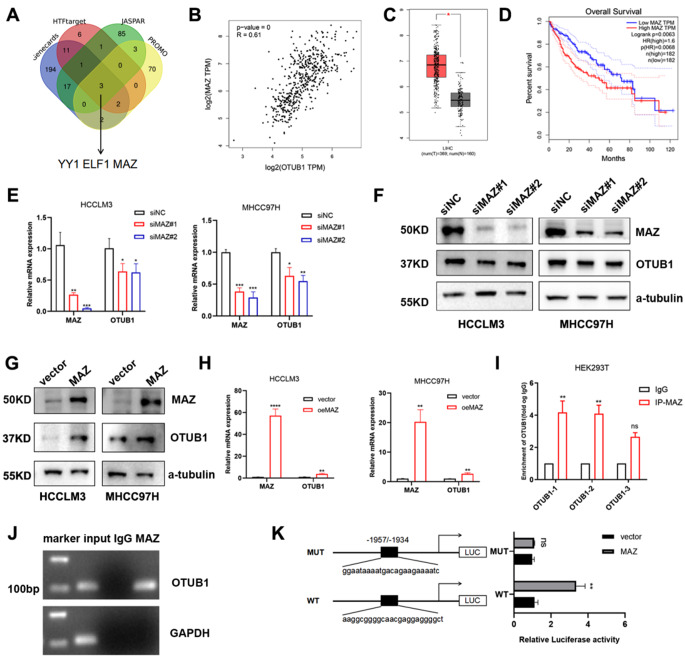
MAZ directly connected to the OTUB1 promoter region to trigger OTUB1 transcription. **a** The Genecards, JASPAR, PROMO, and HTFtarget databases were analyzed by Venn diagram, and 3 candidate transcription factors (YY1, ELF1, and MAZ) were finally confirmed. **b** OTUB1 and MAZ mRNA levels in the TCGA-LIHC database were analyzed by Pearson correlation. **c**, **d** The mRNA expression and the overall survival (OS) curves of MAZ in HCC via the GEPIA database (http://gepia2.cancer-pku.cn/). **e**, **f** qPCR and immunoblotting were conducted to measure the mRNA and protein levels of MAZ and OTUB1 in HCCLM3 and MHCC97H cells transfected with siNC or siMAZ, respectively. **g**, **h** qPCR and immunoblotting were conducted to measure the mRNA and protein levels of MAZ and OTUB1 in HCCLM3 and MHCC97H cells transfected with indicated plasmids, respectively. **i**, **j** ChIP data showed that MAZ binds to OTUB1 in HCCLM3 cells. **k** Dual-luciferase reporter assays showed that MAZ acts on the OTUB1 promoter region in HCCLM3 cells. Data represent means ± SEM. *p < 0.05, **p < 0.01, ***p < 0.001

In an attempt to explore the possible upstream regulators contributing to OTUB1 amplification in HCC, we initially examined the Genecards, JASPAR, PROMO, and HTFtarget databases (Fig. 8a). Bioinformatic analysis revealed a strong correlation between the expression of MAZ and OTUB1 (Fig. 8b), and high levels of MAZ were also associated with an unfavorable prognosis for HCC (Fig. 8c, d), indicating that MAZ might be connected with the regulation of OTUB1 expression. Our experiments showed the mRNA and protein levels of OTUB1 were downregulated by MAZ silencing (Fig. 8e, f), whereas MAZ overexpression had the opposite effects (Fig. 8g, h). However, YY1 or ELF1 disruption displayed negligible effects on the OTUB1 transcription level (results not shown). Additionally, the ChIP assay revealed MAZ directly attached to the hypothesized OTUB1 locations in HCCLM3 cells (Fig. 8i, j). We constructed OTUB1 promoter luciferase reporter vectors with (OTUB1-WT) or without (OTUB1-mut) MAZ-binding motif which is predicted to be located in the DNA sequence spanning from 1934 to 1957 bps above transcription start site by JASPAR. MAZ overexpression stimulated OTUB1-WT promoter-driven luciferase expression, while lost impact in the OTUB1-mut group (Fig. 8k). Collectively, these findings proved that MAZ positively regulated OTUB1 expression in HCC. Finally, we evaluated the correlation of OTUB1 and RACK1 in collected clinical HCC samples. Immunoblotting shows that OTUB1 expression positively correlated with RACK1 expression (r = 0.885, *p* < 0.001) in the collected HCC samples (Fig. S3A, B). Accordingly, IHC analysis of HCC tissues pointed to similar results (Fig. S3C). Moreover, AKT, FAK and ERK phosphorylation levels were higher in OTUB1-high tumor tissues as compared with OTUB1-low tumor tissues. These results indicated that OTUB1 might act as an oncogene by regulating RACK1’s functions in HCC. Collectively, aberrant OTUB1 upregulation may own to MAZ upregulation during HCC genesis and progression. Targeting MAZ-OTUB1-RACK1 is a promised HCC therapy strategy.

## Discussion

The dismal 5-year survival rate of HCC urges for more detailed mechanism research [24]. DUBs were demonstrated to affect the ubiquitination and stability of oncoproteins [25], which ameliorates tumors [10, 26]. However, the roles of DUBs in HCC haven’t been fully elucidated. Overexpression of OTUB1 has been identified to be responsible for the pathogenesis of multiple malignancies, including HCC [27]. Previous reports indicated that OTUB1 regulates solid tumors genesis and progression through numerous signaling pathways through deubiquitinating signaling molecules like YAP, Raptor, DEPTOR, SMAD2/3, Hif-1α, FOXM1, HK2, GPX4, c-Maf, c-IAP, PD-L1, SLC7A11, TRAF6, etc. [15, 17, 21, 28–37]. In accordance with prior research, we discovered that OTUB1 increased HCC proliferative, migratory, and invading activity both in vitro and in vivo. To date, inhibitors that directly and specifically target OTUB1 have not been reported. Henning et al. [38] proposed a deubiquitinase-targeting chimeras (DUBTACs), in which the covalent small molecule recruiting ligand EN523 of OTUB1, could be used as part of a heterobifunctional DUBTAC to stabilize target proteins in cells to exert antitumor effects. Therefore, OTUB1 has the potential to become a novel marker for predicting tumor progression, recurrence, and metastasis, and has broad prospects in the research and development of novel antitumor drugs.

In our study, the LC-MS/MS and immunoprecipitation assays revealed that RACK1 is a novel substrate for OTUB1 in HCC. RACK1 mRNA levels in HCC cells with stable OTUB1 knockdown or overexpression were unaffected, while protein levels altered correspondingly. Knockdown of OTUB1 accelerated the half-life reduction of RACK1 induced by CHX treatment, while MG132 treatment improved the stability of RACK1 in HCC. These suggested that OTUB1 regulated RACK1 expression through post-translational ubiquitination modifications. The in vitro ubiquitination assay demonstrated that OTUB1 blocked K48-linked polyubiquitination of RACK1 via its non-canonical ubiquitination activity by examining the effect of its two mutants with different enzyme activities. Immunoblotting showed that OTUB1 overexpression enhanced RACK1 and led to the activation of the Akt and ERK/FAK pathways in HCC cells. Furthermore, functional phenotype experiments indicated that the inhibitory ability of OTUB1 knockdown on malignant phenotypes and signaling transduction in HCC cells was reversed by RACK1 overexpression. These results suggested that OTUB1 regulated HCC proliferation, metastasis, and progression partly through RACK1.

RACK1 was a member of the Trp-Asp (WD) repeat protein family, which acted as a scaffolding protein for signaling transduction for the development of multiple malignancies. RACK1 exacerbates cancer via activating multiple proto-tumoral processes in other solid tumors [22, 39]. In HCC, ribosomal RACK1 and PKCβII functioned together to stimulate eukaryotic initiation factor 4E (eIF4E) phosphorylation, which triggered preferential translation of the powerful factors necessary for growth and survival [9]. RACK1 was highly O-GlcNAcylation modified at Ser122, which prevents RACK1 from ubiquitination-proteasome degradation and enhances hepatocellular carcinogenesis [8]. Ubiquitination modification is critical for the RACK1 function. The E3 ligase RAB40C ubiquitinates and destabilizes RACK1 to promote cancer cell proliferation [40]. However, the direct deubiquitinase of RACK1 remains unclear in HCC. In our research, we discovered that OTUB1 deubiquinates and stabilizes RACK1 and its expression is abnormally overexpressed after HCC genesis. OTUB1 overexpression significantly accelerated HCC cell proliferation and invasion as RACK1 did; OTUB1 overexpression lost stimulation upon the RACK1-deficient HCC cells. OTUB1 is a unique DUB that has both canonical deubiquitinase activity dependent on the C91 residue and a non-canonical deubiquitinating activity by binding to certain E2 enzymes to prevent Ub chain transfer. In the later condition, the D88 residue is responsible for the interaction between OTUB1 and E2-conjugating enzymes, thereby blocking the synthesis of polyubiquitin chains on target proteins. Thus, we have constructed two mutants: C91A (defective in canonical deubiquitinase activity) and D88A (defective in binding to E2 enzymes), and evaluated their effects in this study. Although the precise mechanism by which OTUB1 induces RACK1 stabilization requires further elucidation, it is very likely that the binding between OTUB1 and RACK1 as well as OTUB1’s ability to inhibit E2-conjugating enzymes recruited by the unknown E3 ligase contribute to RACK1 stabilization induced by OTUB1. This will be the direction of our further work to explore the detailed mechanisms.

Numerous studies also supported the involvement of RACK1 in the regulation of immune response. Previous reports indicated that RACK1 activation was closely associated with inflammasome signaling pathway and the production of the pro-inflammatory cytokines IL-8 and TNF-α [41, 42]. Currently, accumulating evidence indicated RACK1 could regulate tumor immunity by influencing the massive recruitment of macrophages and secretion of proinflammatory cytokines. In gastric cancer, cyclase-associated protein 2 (CAP2) bound to RACK1 and activated the SRC/FAK/ERK signaling pathway, leading to IL-4 and IL10 secretion, and M2 macrophage polarization [43]. In oral squamous cell carcinoma, RACK1 decreased IL-6, CCL5, and CSF levels and increased the M2/M1 ratio in an NF-κB axis-dependent manner [44]. Thus, targeting RACK1 in combination with immunotherapy may be a promising strategy for the treatment of cancer. Therefore, we will explore whether OTUB1 affects the function of macrophages and other immune cells through RACK1 in HCC and its potential related mechanisms in our future studies to improve HCC patient outcomes in cancer immunotherapy.

The transcription factor MAZ, which has a unique zinc finger structure, is abundantly expressed in many malignancies [45]. MAZ was recognized to play a pro-cancer role by exerting the transcriptional regulation effects upon the many genes related to various cellular processes, including the regulation of cell proliferation, EMT, angiogenesis, autophagy, etc. [46–48]. MAZ was highly expressed in HCC, and was positively correlated with advanced clinicopathological characteristics and poor overall survival of HCC patients [49, 50]. As a transcription factor, MAZ cooperates with c-MYC to facilitate the GA box (GGGAGGG)-regulated gene transcription initiation and termination [51]. The heightened activities of MAZ-MYC and dense promoter mutations accentuated activated transcriptional regulation in NAFLD-HCC [52]. In this study, we found MAZ promoted OTUB1 expression by recognizing a putative response element localized on the promoter region of OTUB1. Abnormally highly expressed OTUB1 recognized and prevented RACK1 for ubiquitination-dependent degradation to promote cell proliferation and invasion, and ultimately promoted the progression of HCC. As the epidemiological characteristics of HCC evolve, these findings suggested that the MAZ-OTUB1-RACK1 axis may present great therapeutic potential in NAFLD-HCC.

In summary, our findings suggest that a unique MAZ-OTUB1-RACK1-PI3K/AKT and FAK/ERK axis plays a critical role in the progression of HCC and that blocking this axis may be an advantageous therapeutic strategy for curing HCC.

## Electronic supplementary material

Below is the link to the electronic supplementary material.

Supplementary Material 1

## Data Availability

Full data will be available from the corresponding author upon reasonable request.

## Abbreviations

USP: Ubiquitin-specific protease
OTU: Ovarian tumor protease
DUBs: Deubiquitinases
CYCS: Cytochrome c
c-Maf: MAF bZIP transcription factor
HK2: Hexokinase 2
FOXM1: Forkhead box M1
ECT-2: Epithelial cell transforming 2
Rho: Ras homolog family
ChIP: Chromatin immunoprecipitation
OTUD3: OTU deubiquitinase 3
OTUB2: OTU deubiquitinase, ubiquitin aldehyde binding 2
OTUD7B: OTU deubiquitinase 7B
OTUD1: OTU deubiquitinase 1
co-IP: Co-immunoprecipitation
CRC: Colorectal cancer
E2: Ubiquitin-conjugating enzyme
E3: Ubiquitin ligase
MAZ: Myc-associated zinc-finger protein
YY1: YY1 transcription factor
ELF1: E74 like ETS transcription factor 1
eIF4E: Eukaryotic initiation factor 4E
PKCβII: Protein kinase C beta
RAB40C: SOCS box-containing protein RAR3
YAP: Yes-associated protein
Raptor: Regulatory associated protein of MTOR complex 1
DEPTOR: DEP domain containing MTOR interacting protein
SMAD2/3: SMAD family member 2/3
Hif-1α: Hypoxia inducible factor 1 subunit alpha
GPX4: Glutathione peroxidase 4
c-IAP: Cellular inhibitor of apoptosis
SLC7A11: Solute carrier family 7 member 11
TRAF6: TNF receptor associated factor 6
