# Supplementary material for: OTUB1 accelerates hepatocellular carcinoma by stabilizing RACK1 via its non-canonical ubiquitination
Source: Cell Oncol (Dordr). 2024 Feb 5;47(3):987–1004. doi: 10.1007/s13402-023-00913-7 (PMC11219430; doi:10.1007/s13402-023-00913-7)
Supplement: Supplementary file 1 — Supplementary Material 1 [file 13402_2023_913_MOESM1_ESM.docx]

**Supplementary Information**

OTUB1 Accelerates Hepatocellular Carcinoma by Stabilizing RACK1 via its non-canonical ubiquitination

Liqun Peng^1,2^, Tiangen Wu^1,2^, Yingyi Liu^1,2^, Dongli Zhao^3^ ,Wenzhi He^1,2,3*^,Yufeng Yuan^1,2*^

^1^Department of Hepatobiliary and Pancreatic Surgery, Zhongnan Hospital of Wuhan University, Wuhan, China.

^2^Clinical Medicine Research Center for Minimally Invasive Procedure of Hepatobiliary & Pancreatic Diseases of Hubei Province, Wuhan, China.

^3^College of Life Sciences, Hubei Key Laboratory of Cell Homeostasis, Wuhan University, Wuhan, China.

*** Correspondence: Yufeng Yuan,** [yuanyf1971@whu.edu.cn;](mailto:yuanyf1971@whu.edu.cn;) **Wenzhi He,** hewz@whu.edu.cn.


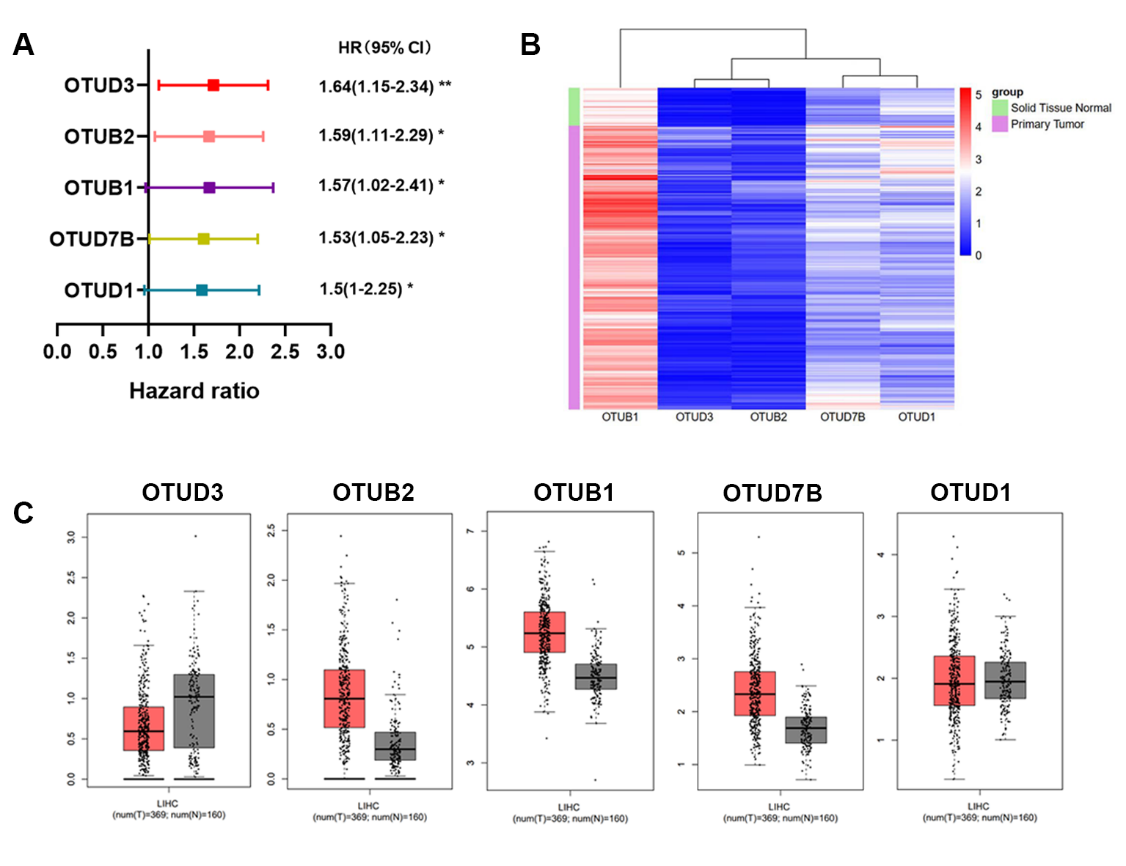


**Supplementary Fig1. The association of OTUs with patient outcomes in HCC**

A. Forest plot showing the HR values with 95% confidence intervals of OTUs retrieved from the Kaplan-Meier Plotter database. B Heatmap showing the expression levels of indicated OTUs in HCC tumor and normal tissues according to the TCGA database. C. The expression levels of indicated OTUs in HCC tumor and normal tissues according to GEPIA database. Data represent means ± SEM. *p < 0.05, **p < 0.01, ***p < 0.001.


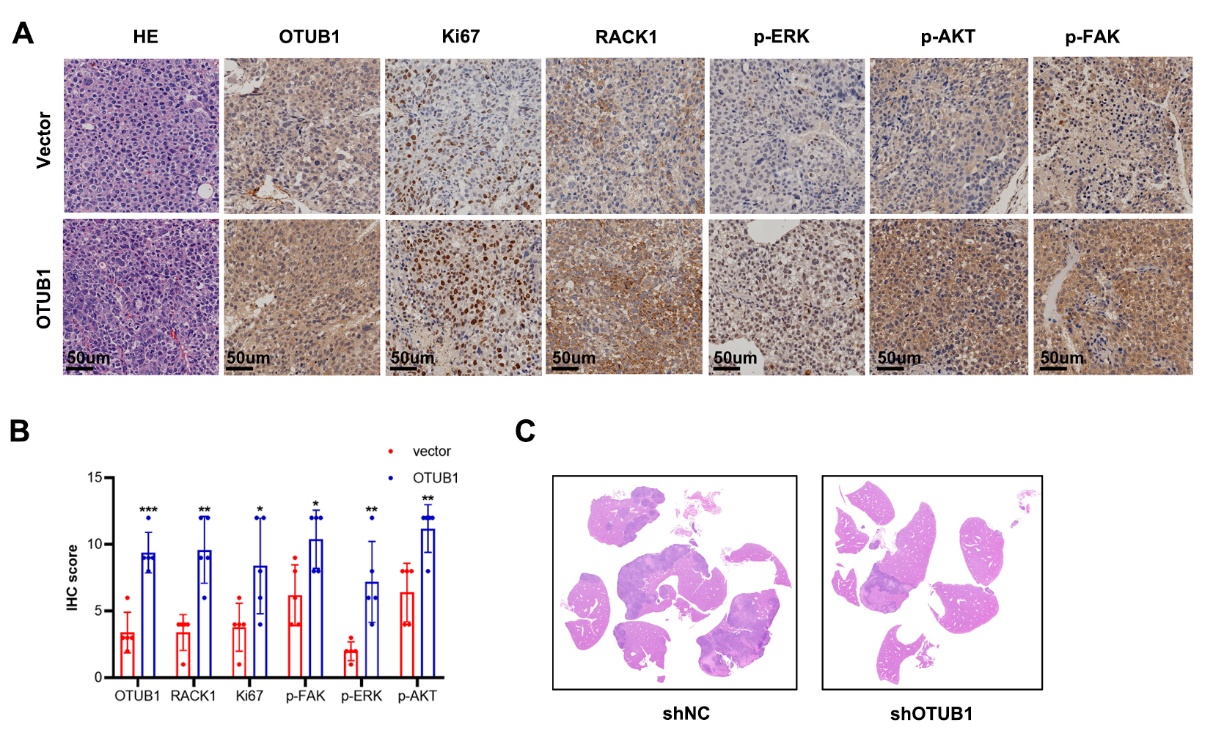


**Supplementary Figure 2. Effect of OTUB1 on HCC growth and metastasis in vivo**

A. IHC staining was performed to determine the protein levels of OTUB1, RACK1, Ki67, p-FAK, p-AKT, and p-ERK in the huh7 xenograft tumors. B.Quantitative IHC scoring of the huh7 xenograft tumors(n=5). C. Representative H&E staining images of orthotopic liver tumor model(n = 3 mice/group).


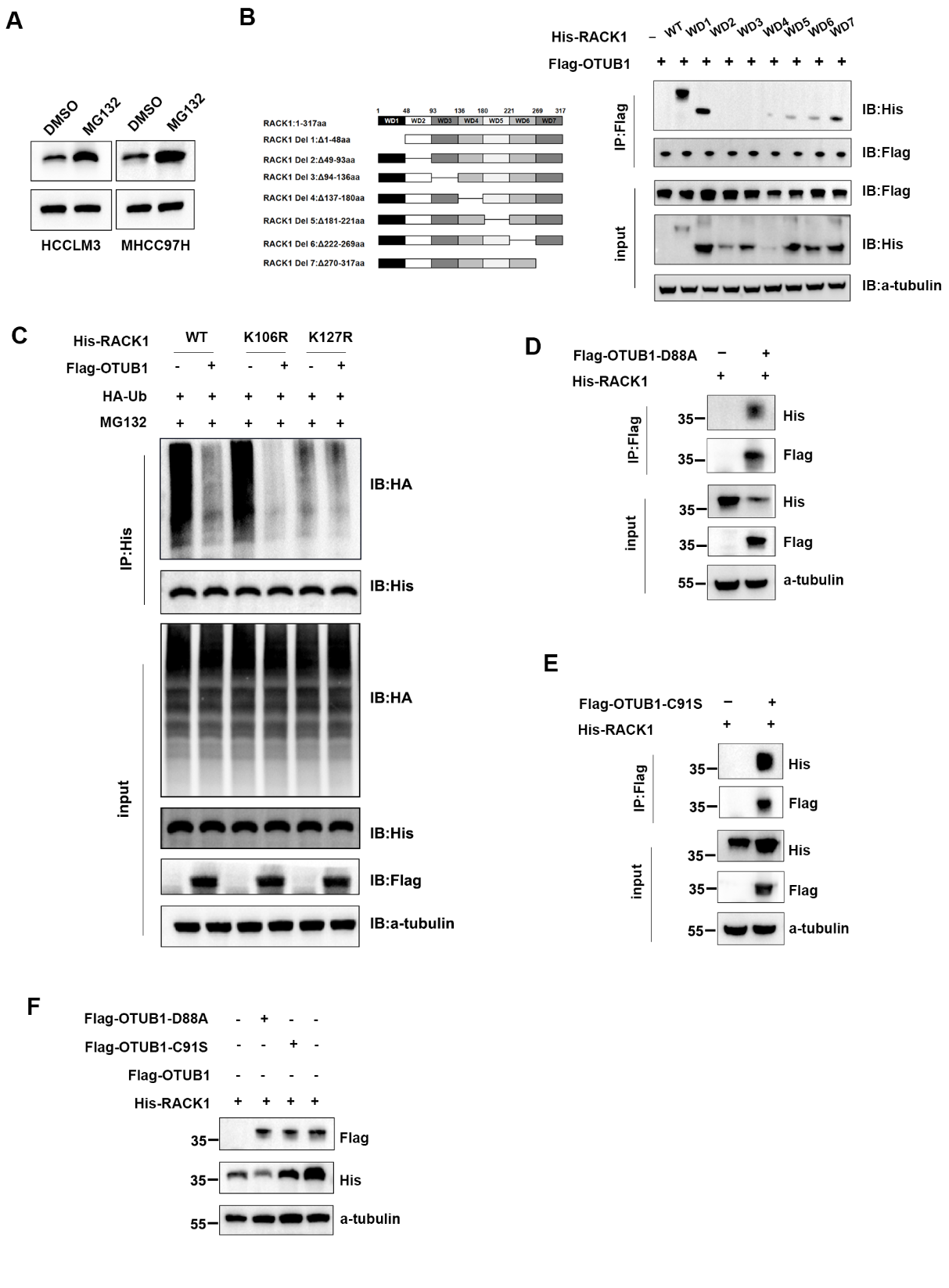


**Supplementary Figure 3. OTUB1 inhibits the ubiquitination of RACK1 dependent of its non-canonical activity**

A. Western blot analysis of RACK1 protein in HCCLM3 and MHCC97H cells. Cells were treated with 10μM MG132 for 8 h before harvest. B. HEK293T cells were transfected with the Flag-OTUB1 plasmids along with the truncated variants of His-RACK1 for 48h. Co-IP assay was then conducted to identify the OTUB1 and RACK1 interaction domains. C. Ubiquitination assay of RACK1 in HEK293T cells co-transfected with HA-Ub, Flag-OTUB1, His-RACK1-WT, His-RACK1-K106R and His-RACK1- K127R and treated with 10 μM MG132 for 8 h. Cell lysates were analyzed by immunoprecipitation with anti-His and western immunoblotting with indicated antibodies. D. Co-immunoprecipitation of Flag-OTUB1-D88A with His-RACK1. HEK293T cells were co-transfected with indicated plasmids for 48 h. Anti-Flag antibody-conjugated agarose beads were used for immunoprecipitation, and the interaction was detected by immunoblotting with the indicated antibodies. E. Co-immunoprecipitation of Flag-OTUB1-C91A with His-RACK1. HEK293T cells were co-transfected with indicated plasmids for 48 h. Anti-Flag antibody-conjugated agarose beads were used for immunoprecipitation, and the interaction was detected by immunoblotting with the indicated antibodies. F. Western-blot of His-RACK1 expression in HEK293T cells transfected with Flag-OTUB1 plasmid or its enzymatically deficient mutants.


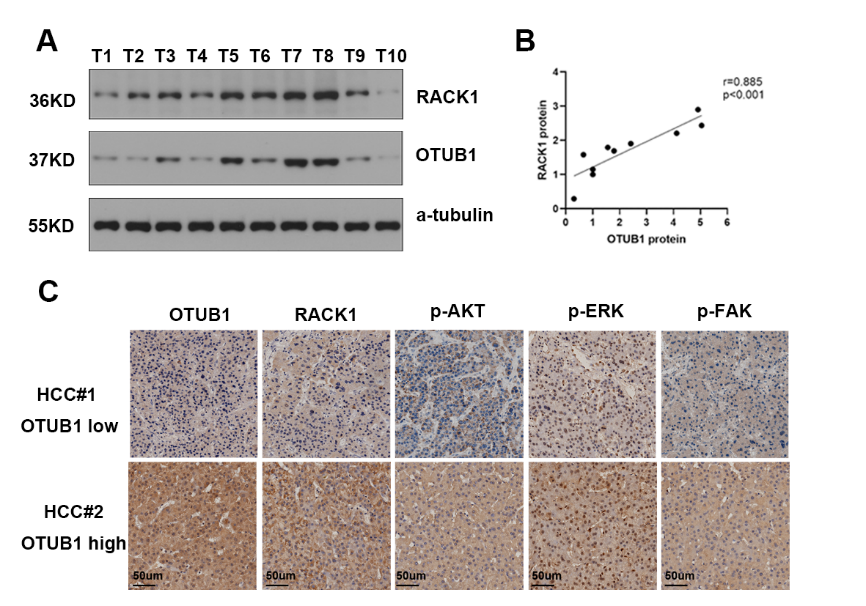


**Supplementary Fig4. OTUB1 expression correlates positively with RACK1 expression in HCC patient samples**

A. Immunoblotting analysis of OTUB1 and RACK1 proteins in clinical samples of HCC tissues(n=10). B. Spearman’s correlation analysis further showed their positive correlation. C. Representative images of IHC staining of OTUB1, RACK1 p-FAK, p-AKT, and p-ERK in HCC patient tissues. Scale bar:200 μm. Data represent means ± SEM. *p < 0.05, **p < 0.01, ***p < 0.001.
